# Residual Multiparticle Entropy for a Fractal Fluid of Hard Spheres

**DOI:** 10.3390/e20070544

**Published:** 2018-07-23

**Authors:** Andrés Santos, Franz Saija, Paolo V. Giaquinta

**Affiliations:** 1 Departamento de Física and Instituto de Computación Científica Avanzada (ICCAEx), Universidad de Extremadura, E-06006 Badajoz, Spain; 2 CNR-IPCF, Viale F. Stagno d’Alcontres, 37-98158 Messina, Italy; 3 Dipartimento di Scienze Matematiche e Informatiche, Scienze Fisiche e Scienze della Terra, Università degli Studi di Messina, Contrada Papardo, 98166 Messina, Italy

**Keywords:** residual multiparticle entropy, hard spheres, fractal dimension

## Abstract

The residual multiparticle entropy (RMPE) of a fluid is defined as the difference, Δs, between the excess entropy per particle (relative to an ideal gas with the same temperature and density), $s_{\mathrm{ex}}$, and the pair-correlation contribution, $s_{2}$. Thus, the RMPE represents the net contribution to $s_{\mathrm{ex}}$ due to spatial correlations involving three, four, or more particles. A heuristic “ordering” criterion identifies the vanishing of the RMPE as an underlying signature of an impending structural or thermodynamic transition of the system from a less ordered to a more spatially organized condition (freezing is a typical example). Regardless of this, the knowledge of the RMPE is important to assess the impact of non-pair multiparticle correlations on the entropy of the fluid. Recently, an accurate and simple proposal for the thermodynamic and structural properties of a hard-sphere fluid in fractional dimension 1<d<3 has been proposed (Santos, A.; López de Haro, M. *Phys. Rev. E* **2016**, *93*, 062126). The aim of this work is to use this approach to evaluate the RMPE as a function of both *d* and the packing fraction ϕ. It is observed that, for any given dimensionality *d*, the RMPE takes negative values for small densities, reaches a negative minimum $\Delta s_{\min}$ at a packing fraction $\phi_{\min}$, and then rapidly increases, becoming positive beyond a certain packing fraction $\phi_{0}$. Interestingly, while both $\phi_{\min}$ and $\phi_{0}$ monotonically decrease as dimensionality increases, the value of $\Delta s_{\min}$ exhibits a nonmonotonic behavior, reaching an absolute minimum at a fractional dimensionality d≃2.38. A plot of the scaled RMPE $\Delta s/|\Delta s_{\min}|$ shows a quasiuniversal behavior in the region $-0.14≲\phi -\phi_{0}≲0.02$.

## 1. Introduction

The properties of liquids are of great interest in many science and engineering areas. Aside from ordinary three-dimensional systems, many interesting phenomena do also occur in restricted one- or two-dimensional geometries, under the effect of spatial confinement. Actually, there are also cases where the configuration space exhibits, at suitable length scales, non-integer dimensions. Indeed, many aggregation and growth processes can be described quite well by resorting to the concepts of fractal geometry. This is the case, for example, of liquids confined in porous media or of assemblies of small particles forming low-density clusters and networks [1,2,3,4].

Heinen et al. [5] generalized this issue by introducing fractal particles in a fractal configuration space. In their framework, the particles composing the liquid are fractal as is the configuration space in which such objects move. Santos and López de Haro [6] have further developed reliable heuristic interpolations for the equation of state and radial distribution function of hard-core fluids in fractal dimensions between one and three dimensions. Taking advantage of their work, we intend to study in this paper some thermostatistical properties of such fractal systems in the theoretical framework provided by the multiparticle correlation expansion of the entropy.

According to the first and second principles of thermodynamics, the entropy per particle s(ρ,β) (in units of the Boltzmann constant $k_{B}$) is defined by the differential relation $ds\left(\rho ,\beta \right)=\beta du\left(\rho ,\beta \right)+\beta p\left(\rho ,\beta \right)d\rho^{-1}$, where ρ is the number density, $\beta =1/k_{B}T$ is the inverse temperature, u(ρ,β) is the internal energy per particle, and p(ρ,β) is the pressure. The excess entropy per particle is

(1) $$s_{\mathrm{ex}}\left(\rho ,\beta \right)=s\left(\rho ,\beta \right)-s_{\mathrm{id}}\left(\rho ,\beta \right),$$

where

(2) $$s_{\mathrm{id}}\left(\rho ,\beta \right)=\frac{d+2}{2}-\ln\left[\rho {\left(\frac{h^{2}\beta }{2\pi m}\right)}^{d/2}\right]$$

is the ideal-gas entropy. In Equation (2), *d* is the spatial dimensionality of the system, *h* is Planck’s constant, and *m* is the mass of a particle. (To keep the presentation as general as possible, along this section and also in Section 2.1, β is included as an argument of the physical quantities. However, starting from Section 2.2, the most general, temperature-dependent case is laid out, and we focus on the particular system studied in the paper, which is athermal.)

As is well known, the excess entropy can be expressed as an infinite sum of contributions associated with spatially integrated density correlations of increasing order [7,8]. In the absence of external fields, the leading and quantitatively dominant term of the series is the so-called “pair entropy”,

(3) $$s_{2}\left(\rho ,\beta \right)=-\frac{\rho }{2}\int dr\;\left[g(r;\rho ,\beta )\ln g(r;\rho ,\beta )-g(r;\rho ,\beta )+1\right],$$

whose calculation solely requires the knowledge of the radial distribution function of the fluid, g(r;ρ,β), which is defined by the identity $n_{2}\left(r_{1},r_{2};\rho ,\beta \right)=\rho^{2}g(|r_{1}-r_{2}\left|;\rho ,\beta \right)$, where $n_{2}\left(r_{1},r_{2};\rho ,\beta \right)$ is the pair correlation function. An integrated measure of the importance of more-than-two-particle density correlations in the overall entropy balance is given by the so-called “residual multiparticle entropy” (RMPE) [9]:

(4) $$\Delta s\left(\rho ,\beta \right)=s_{\mathrm{ex}}\left(\rho ,\beta \right)-s_{2}\left(\rho ,\beta \right).$$

It is important to note that, at variance with $s_{\mathrm{ex}}$ and $s_{2}$, which are both negative definite quantities, Δs may be either negative or positive. As originally shown by Giaquinta and Giunta for hard spheres in three dimensions [9], the sign of this latter quantity does actually depend on the thermodynamic state of the fluid. In fact, the RMPE of a hard-sphere fluid is negative at low densities, thus contributing to a global reduction of the phase space available to the system as compared to the corresponding ideal gas. However, the RMPE undergoes a sharp crossover from negative to positive values at a value of the packing fraction which substantially overlaps with the thermodynamic freezing threshold of the hard-sphere fluid. Such a behavior suggests that at high enough densities multiparticle correlations play an opposite role with respect to that exhibited in a low packing regime in that they temper the decrease of the excess entropy that is largely driven by the pair entropy. The change of sign presented by the RMPE is a background indication, intrinsic to the fluid phase, that particles, forced by more and more demanding packing constraints, start exploring, on a local scale, a different structural condition. This process is made possible by an increasing degree of cooperativity that is signaled by the positive values attained by Δs, which gradually leads to a more efficacious spatial organization and ultimately triggers the crystalline ordering of the system on a global scale.

A similar indication is also present in the RMPE of hard rods in one dimension [10]. In this model system, notwithstanding the absence of a fluid-to-solid transition, one can actually observe the emergence of a solid-like arrangement at high enough densities: tightly-packed particles spontaneously confine themselves within equipartitioned intervals whose average length is equal to the the total length per particle, even if the onset of a proper entropy-driven phase transition is frustrated by topological reasons. Again, even in this “pathological” case, the vanishing of the RMPE shows up as an underlying signature of a structural change which eventually leads to a more ordered arrangement.

The relation between the zero-RMPE threshold and the freezing transition of hard spheres apparently weakens in four and five dimensions [11], where lower bounds of the entropy threshold significantly overshoot the currently available computer estimates of the freezing density [11,12]. In fact, Krekelberg et al. [11,12] suggested the possibility that high-dimensional hard-sphere fluids may even encounter the glass transition upon densification before reaching the zero-RMPE point.

On the other side, a close correspondence between the sign crossover of the RMPE and structural or thermodynamical transition thresholds has been highlighted in both two and three dimensions on a variety of model systems for different macroscopic ordering phenomena other than freezing [13], including fluid demixing [14], the emergence of mesophases in liquid crystals [15], the formation of a hydrogen-bonded network in water [16], or, more recently, the onset of glassy dynamics [17].

If hard-core systems in fractal geometries exhibit a sort of disorder-to-order transition, it seems plausible that such a transition is signaled by a change of sign of Δs. Taking all of this into account, it is desirable to study the RMPE of hard-core fractal fluids, and this is the main goal of this paper. It is organized as follows. The theoretical approach of Ref. [6] is described and applied to the evaluation of the RMPE in Section 2. The results are presented and discussed in Section 3. Finally, the main conclusions of the work are recapped in Section 4.

## 2. Methods

### 2.1. General Relations

In principle, the knowledge of the radial distribution function, g(r;ρ,β), allows one to determine the pair entropy from Equation (3). This is equivalent to

(5) $$s_{2}\left(\rho ,\beta \right)=\frac{1}{2}\left[\chi_{T}\left(\rho ,\beta \right)-1\right]+{\tilde{s}}_{2}\left(\rho ,\beta \right),$$

where

(6) $$\chi_{T}\left(\rho ,\beta \right)=1+\rho \int dr\;\left[g(r;\rho ,\beta )-1\right]$$

is the isothermal susceptibility and we have called

(7) $${\tilde{s}}_{2}\left(\rho ,\beta \right)=-\frac{\rho }{2}\int dr\;g\left(r;\rho ,\beta \right)\ln g\left(r;\rho ,\beta \right).$$

Thus, Equation (4) can be rewritten as

(8) $$\Delta s\left(\rho ,\beta \right)=s_{\mathrm{ex}}\left(\rho ,\beta \right)-\frac{1}{2}\left[\chi_{T}\left(\rho ,\beta \right)-1\right]-{\tilde{s}}_{2}\left(\rho ,\beta \right).$$

Equations (5)–(8) hold regardless of whether the total potential energy $U(r_{1},r_{2},r_{3},\ldots )$ is pairwise additive or not. On the other hand, if *U* is pairwise additive, the knowledge of g(r;ρ,β) yields, apart from $s_{2}\left(\rho ,\beta \right)$, the thermodynamic quantities of the system via the so-called thermodynamic routes [18]. In particular, the virial route is

(9) $$\begin{array}{cc} Z\left(\rho ,\beta \right)\equiv \frac{\beta p(\rho ,\beta )}{\rho } & =1-\frac{\rho \beta }{2d}\int dr\;r\frac{d\varphi (r)}{dr}g\left(r;\rho ,\beta \right)\\ & =1+\frac{\rho }{2d}\int dr\;r\frac{de^{-\beta \varphi (r)}}{dr}y\left(r;\rho ,\beta \right), \end{array}$$

where *Z* is the compressibility factor, φ(r) is the pair interaction potential, and $y\left(r;\rho ,\beta \right)\equiv \;e^{\beta \varphi (r)}g\left(r;\rho ,\beta \right)$ is the so-called cavity function. Next, the excess Helmholtz free energy per particle, $a_{\mathrm{ex}}$, and the excess entropy per particle, $s_{\mathrm{ex}}$, can be obtained by standard thermodynamic relations as

(10) $$\beta a_{\mathrm{ex}}\left(\rho ,\beta \right)=\int_{0}^{1}dt\frac{Z(\rho t,\beta )-1}{t},\;s_{\mathrm{ex}}\left(\rho ,\beta \right)=\beta \frac{\partial \beta a_{\mathrm{ex}}\left(\rho ,\beta \right)}{\partial \beta }-\beta a_{\mathrm{ex}}\left(\rho ,\beta \right).$$

Combining Equations (9) and (10), we obtain

(11) $$s_{\mathrm{ex}}\left(\rho ,\beta \right)=\frac{\rho }{2d}\left(\beta \frac{\partial }{\partial \beta }-1\right)\int dr\;r\frac{de^{-\beta \varphi (r)}}{dr}\int_{0}^{1}dt\;y\left(r;\rho t,\beta \right).$$

To sum up, assuming the radial distribution function g(r;ρ,β) for a *d*-dimensional fluid of particles interacting via an interaction potential φ(r) is known, it is possible to determine the excess entropy (see Equation (1)), the pair entropy (see Equation (3)), and hence the RMPE Δs. Note that, while $s_{2}$ only requires g(r) at the state point (ρ,β) of interest, $s_{\mathrm{ex}}$ requires the knowledge of g(r) at all densities smaller than ρ and at inverse temperatures in the neighborhood of β.

A remark is now in order. The isothermal susceptibility $\chi_{T}\left(\rho ,\beta \right)$ can be obtained directly from g(r;ρ,β) via Equation (6) or indirectly via Equation (9) and the thermodynamic relation

(12) $$\chi_{T}^{-1}\left(\rho ,\beta \right)=\frac{\partial \rho Z(\rho ,\beta )}{\partial \rho }.$$

If the correlation function g(r;ρ,β) is determined from an approximate theory, the compressibility route in Equation (6) and the virial route given by Equations (9) and (12) yield, in general, different results.

### 2.2. Fractal Hard Spheres

Now, we particularize to hard-sphere fluids in *d* dimensions. The interaction potential is simply given by

(13) $$\varphi \left(r\right)=\left\{\begin{array}{cc} \infty , & r<\sigma ,\\ 0, & r>\sigma , \end{array}\right.$$

where σ is the diameter of a sphere. In this case, the radial distribution function g(r;ϕ) is independent of temperature and the density can be characterized by the packing fraction

(14) $$\phi \equiv \frac{{\left(\pi /4\right)}^{d/2}}{\Gamma (1+d/2)}\rho \sigma^{d}.$$

Taking into account that $\frac{d}{dr}e^{-\beta \varphi (r)}=\delta \left(r-\sigma \right)$, Equations (9) and (11) become

(15) $$Z\left(\phi \right)=1+2^{d-1}\phi g_{c}\left(\phi \right),$$

(16) $$s_{\mathrm{ex}}\left(\phi \right)=-\beta a_{\mathrm{ex}}\left(\phi \right)=2^{d-1}\phi \int_{0}^{1}dt\;g_{c}\left(\phi t\right),$$

where $g_{c}\left(\phi \right)=g\left(\sigma^{+};\phi \right)=y\left(\sigma ;\phi \right)$ is the *contact* value of the radial distribution function. In addition, Equation (7) can be written as

(17) $${\tilde{s}}_{2}\left(\phi \right)=-d2^{d-1}\phi \int_{0}^{\infty }dr\;r^{d-1}g\left(r;\phi \right)\ln g\left(r;\phi \right).$$

In Equations (14)–(17), it is implicitly assumed that *d* is an integer dimensionality. However, in a pioneering work, Heinen et al. [5] introduced the concept of classical liquids in fractal dimension and performed Monte Carlo (MC) simulations of fractal “spheres” in a fractal configuration space, both with the same noninteger dimension. Such a generic model of fractal liquids can describe, for instance, microphase separated binary liquids in porous media and highly branched liquid droplets confined to a fractal polymer backbone in a gel. For a discussion on the use of two-point correlation functions in fractal spaces, see Ref. [19].

It seems worthwhile extending Equations (14)–(17) to a non-integer dimension *d* and studying the behavior of the RMPE Δs as a function of both ϕ and *d*. To this end, an approximate theory providing the radial distribution function g(r;ϕ) for non-integer *d* is needed. In Ref. [5], Heinen et al. solved numerically the Ornstein–Zernike relation [20] by means of the Percus–Yevick (PY) closure [21]. However, since one needs to carry out an integration in Equation (17) over all distances, an analytic approximation for g(r;ϕ) seems highly desirable.

In Ref. [6], a simple analytic approach was proposed for the thermodynamic and structural properties of the fractal hard-sphere fluid. Comparison with MC simulation results for d=1.67659 showed results comparable to or even better than those obtained from the numerical solution of the PY integral equation. In this approach the contact value of the radial distribution function is approximated by

(18) $$g_{c}\left(\phi \right)=\frac{1-k_{d}\phi }{{\left(1-\phi \right)}^{2}},$$

with

(19) $$k_{d}=\frac{\left(5-d)(2-d\right)}{4}+\left(3-d\right)\left(d-1\right)k_{2},\;k_{2}=\frac{2\sqrt{3}}{\pi }-\frac{2}{3}≃0.436.$$

When particularized to d=1, 2 and 3, Equation (18) gives the exact [18], the Henderson [22], and the PY [23,24] results, respectively. Insertion into Equation (15) gives the compressibility factor Z(ϕ) and, by application of Equation (12), the isothermal susceptibility as

(20) $$\chi_{T}\left(\phi \right)={\left[1+2^{d-1}\phi \frac{2-k_{d}\phi \left(3-\phi \right)}{{\left(1-\phi \right)}^{3}}\right]}^{-1}.$$

Note that $2-k_{d}\phi \left(3-\phi \right)\geq 0$ for all d≥1 and 0≤ϕ≤1, so that $\chi_{T}\left(\phi \right)$ is mathematically well defined. Analogously, Equation (16) yields

(21) $$s_{\mathrm{ex}}\left(\phi \right)=-2^{d-1}\left[\frac{(1-k_{d})\phi }{1-\phi }-k_{d}\ln\left(1-\phi \right)\right].$$

Thus, to complete the determination of Δs from Equation (8), only ${\tilde{s}}_{2}$ remains. It requires the knowledge of the full radial distribution function (see Equation (17)). In the approximation of Ref. [6], g(r;ϕ) is given by the simple interpolation formula

(22) $$g\left(r;\phi \right)=\alpha \left(\phi \right)g_{1D}\left(r;\phi_{1D}^{\mathrm{eff}}\left(\phi \right)\right)+\left[1-\alpha \left(\phi \right)\right]g_{3D}\left(r;\phi_{3D}^{\mathrm{eff}}\left(\phi \right)\right),$$

where $g_{1D}\left(r;\phi \right)$ and $g_{3D}\left(r;\phi \right)$ are the exact and PY functions for d=1 and 3, respectively,

(23) $$\phi_{1D}^{\mathrm{eff}}\left(\phi \right)=\frac{g_{c}\left(\phi \right)-1}{g_{c}\left(\phi \right)},\;\phi_{3D}^{\mathrm{eff}}\left(\phi \right)=\frac{1+4g_{c}\left(\phi \right)-\sqrt{1+24g_{c}\left(\phi \right)}}{4g_{c}\left(\phi \right)}$$

are effective packing fractions, and

(24) $$\alpha \left(\phi \right)=\frac{H\left(\phi \right)-H_{3D}\left(\phi_{3D}^{\mathrm{eff}}\left(\phi \right)\right)}{H_{1D}\left(\phi_{1D}^{\mathrm{eff}}\left(\phi \right)\right)-H_{3D}\left(\phi_{3D}^{\mathrm{eff}}\left(\phi \right)\right)}$$

is the mixing parameter. In Equation (24),

(25) $$H\left(\phi \right)=\frac{\frac{1}{2}-A_{d}\phi +C_{d}\phi^{2}}{1+\left(d-1\right)\phi \left[1+\left(3-d\right)\left(1-2k_{2}\right)\left(3-\phi \right)\phi \right]},$$

with

(26) $$A_{d}=\frac{\left(2-d)(63-23d\right)}{60}+\frac{3(d-1)(3-d)}{4}k_{2},\;C_{d}=\frac{\left(2-d)(8-3d\right)}{20}+\frac{\left(d-1)(3-d\right)}{4}k_{2}.$$

Of course, $H_{1D}\left(\phi \right)$ and $H_{3D}\left(\phi \right)$ are obtained from Equation (25) by setting d=1 and d=3, respectively.

Summing up, the proposal of Ref. [6] for noninteger *d* is defined by Equations (22)–(24), with $g_{c}\left(\phi \right)$ and H(ϕ) being given by Equations (18) and (25), respectively. By construction, this approximation reduces to the exact and PY results in the limits d→1 and d→3, respectively. Moreover, it is consistent (via both the virial and compressibility routes) with Henderson’s equation of state [22] in the limit d→2. The corresponding isothermal susceptibility and excess free energy are given by Equations (20) and (21). Finally, Δs(ϕ) can be obtained from Equation (8) by evaluating ${\tilde{s}}_{2}\left(\phi \right)$ from Equation (17) numerically. To that end, and to avoid finite-size effects, it is convenient to split the integration range 0<r<∞ into 0<r<R and R<r<∞, with R=10σ. In the first integral, the analytically known function g(r;ϕ) is used, while, in the second integral, g(r;ϕ) is replaced by its asymptotic form [6].

## 3. Results and Discussion

**Figure 1:**
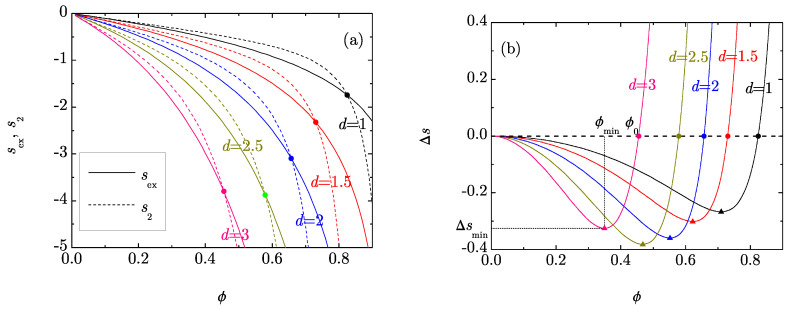
(**a**) Plot of $s_{\mathrm{ex}}\left(\phi \right)$ (solid lines) and $s_{2}\left(\phi \right)$ (dashed lines) for dimensions d=1, 1.5, 2, 2.5 and 3. The circles indicate the points where $s_{\mathrm{ex}}\left(\phi \right)$ and $s_{2}\left(\phi \right)$ cross; (**b**) Plot of $\Delta s\left(\phi \right)=s_{\mathrm{ex}}\left(\phi \right)-s_{2}\left(\phi \right)$ for d=1, 1.5, 2, 2.5 and 3. The triangles indicate the location of the minima and the circles indicate the packing fractions $\phi_{0}$ where Δs=0.

Figure 1a shows $s_{\mathrm{ex}}\left(\phi \right)$ and $s_{2}\left(\phi \right)$ as functions of the packing fraction for a few dimensions 1≤d≤3. In all cases, both functions become more negative as the packing fraction increases. Moreover, at a common packing fraction ϕ, both $s_{\mathrm{ex}}\left(\phi \right)$ and $s_{2}\left(\phi \right)$ decrease as the dimensionality increases. This is an expected property in the conventional case of integer *d* since, at a common ϕ, all the thermodynamic quantities depart more from their ideal-gas values with increasing *d*. Not surprisingly, this property is maintained in the case of non-integer *d*.

Figure 1a also shows that the pair entropy $s_{2}\left(\phi \right)$ overestimates the excess entropy $s_{\mathrm{ex}}\left(\phi \right)$ for packing fractions smaller than a certain value $\phi_{0}$. This means that, if $\phi <\phi_{0}$, the cumulated effect of correlations involving three, four, five, etc. particles produces a decrease of the entropy. The opposite situation occurs, however, if $\phi >\phi_{0}$. At the threshold point $\phi =\phi_{0}$, the cumulated effect of multiparticle correlations cancels and then only the pair correlations contribute to $s_{\mathrm{ex}}$.

The density dependence of the RMPE $\Delta s=s_{\mathrm{ex}}-s_{2}$ is shown in Figure 1b for the same values of *d* as in Figure 1a. The qualitative shape of Δs(ϕ) is analogous for all *d*: Δs starts with a zero value at ϕ=0, then decreases as a convex function, changes its curvature at a given inflection point [9], and reaches a minimum value $\Delta s_{\min}$ at a certain packing fraction $\phi_{\min}$, after which it grows very rapidly, crossing the zero value at the packing fraction $\phi_{0}$.

**Figure 2:**
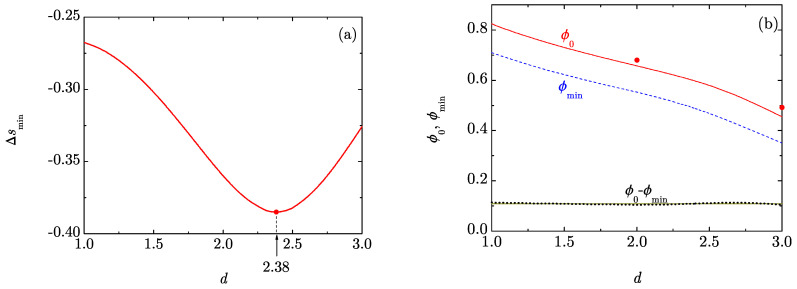
(**a**) Plot of $\Delta s_{\min}$ as a function of *d*. The circle and the arrow indicate the location of the minimum at d≃2.38; (**b**) Plot of $\phi_{0}$ (solid line), $\phi_{\min}$ (dashed line), and the difference $\phi_{0}-\phi_{\min}$ (dotted line) as functions of *d*. The horizontal solid line signals the value $\phi_{0}-\phi_{\min}=0.109$. The circles represent the values ϕ=0.68 at d=2 and ϕ=0.49 at d=3 corresponding to the fluid-hexatic [25,26] and fluid-crystal [27,28,29] transitions, respectively.

The dimensionality dependence of the minimum value of the RMPE, $\Delta s_{\min}$, is displayed in Figure 2a. Interestingly, as can also be observed in Figure 1a, $\Delta s_{\min}$ presents a nonmonotonic variation with *d*, having an absolute minimum $\Delta s_{\min}≃-0.385$ at d≃2.38. At this non-integer dimensionality, the pair entropy $s_{2}$ represents the largest overestimate of the excess entropy $s_{\mathrm{ex}}$. In contrast to $\Delta s_{\min}$, both $\phi_{0}$ and $\phi_{\min}$ decay monotonically with increasing *d*. This is clearly observed in Figure 2b, where also the fluid-hexatic and the fluid-crystal transition points for disks and spheres, respectively, are shown. The proximity of those two points to the curve $\phi_{0}$ provide support to the zero-RMPE criterion, especially considering the approximate character of our simple theoretical approach. Thus, if a disorder-to-order transition phase is possible for fractal hard-core liquids, we expect that it is located near (possibly slightly above) the packing fraction $\phi_{0}$.

**Figure 3:**
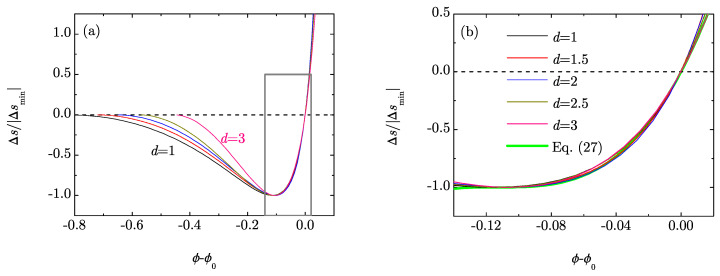
(**a**) Plot of the scaled RMPE $\Delta s/|\Delta s_{\min}|$ as a function of the difference $\phi -\phi_{0}$ for dimensions d=1, 1.5, 2, 2.5 and 3; (**b**) Magnification of the framed region of (**a**). The light thick line represents the formula given by Equation (27).

An interesting feature of Figure 2b is that the difference $\phi_{0}-\phi_{\min}≃0.109$ is hardly dependent on *d*. This suggests the possibility of a quasi-universal behavior of the *scaled* RMPE $\Delta s/|\Delta s_{\min}|$ in the neighborhood of $\phi =\phi_{0}$. To check this possibility, Figure 3a shows $\Delta s/|\Delta s_{\min}|$ as a function of $\phi -\phi_{0}$ for the same dimensionalities as in Figure 1. We can observe a relatively good collapse of the curves in the region $-0.14≲\phi -\phi_{0}≲0.02$. A magnification of that region is shown in Figure 3b. A simple fit can be obtained as follows. Let us define $X\equiv (\phi -\phi_{0})/0.109$ and $Y\left(X\right)\equiv \Delta s\left(\phi \right)/|\Delta s_{\min}|$. Then, a cubic function Y(X) consistent with the conditions Y(0)=0, Y(−1)=−1, $Y^{\prime }\left(-1\right)=0$, $Y^{\prime \prime }\left(-1\right)>0$ is $Y\left(X\right)=X[2+X+c{\left(1+X\right)}^{2}]$ with c<1. A good agreement is found with 0.8<c<1 and we choose c=0.9. In summary, our proposed universal form is

(27) $$\frac{\Delta s(\phi )}{|\Delta s_{\min}|}≃X\left[2+X+c{\left(1+X\right)}^{2}\right],\;X\equiv \frac{\phi -\phi_{0}}{0.109},\;c=0.9.$$

It is also plotted in Figure 3b, where we can see that it captures well the behavior for dimensions 1≤d≤3.

Before closing this section, it is convenient to add a comment. As said at the end of Section 2, the values of Δs have been obtained from Equation (8) by evaluating ${\tilde{s}}_{2}$ from Equation (17) numerically. Since in Equation (20) we have followed the virial route, here we refer to this method to obtain the function Δs as the virial route and denote the resulting quantity as $\Delta s^{\mathrm{vir}}$. On the other hand, this method is not exactly equivalent to that obtained from Equation (1) with $s_{2}$ evaluated numerically from Equation (3) by following the same procedure as described above for ${\tilde{s}}_{2}$. This alternative method is referred to as the compressibility route ($\Delta s^{\mathrm{comp}}$), since it is equivalent to evaluating the isothermal compressibility from Equation (6). Therefore, according to Equation (8),

(28) $$\Delta s^{\mathrm{vir}}-\Delta s^{\mathrm{comp}}=-\frac{1}{2}\left(\chi_{T}^{\mathrm{vir}}-\chi_{T}^{\mathrm{comp}}\right).$$

We have checked that both methods (virial and compressibility) yield practically indistinguishable results. For instance, if d=3, $\phi_{0}=0.4552$ in the virial route, while $\phi_{0}=0.4547$ in the compressibility route. At d=1 and d=2, both methods yield, consistently, $\phi_{0}=0.8246$ and $\phi_{0}=0.6573$, respectively. Note that the compressibility route to measure Δs still has a virial “relic” in the contribution coming from the excess free energy (Equation (21)). A pure compressibility route would require the numerical evaluation of $\chi_{T}$ from Equation (6) and then a double numerical integration, as evident from Equations (10) and (12). This procedure would complicate enormously the evaluation of $s_{\mathrm{ex}}$ without any significant gain in accuracy.

## 4. Conclusions

In this article, we have calculated the pair contribution and the cumulative contribution arising from correlations involving more than two particles to the excess entropy of hard spheres in fractional dimensions 1<d<3. To this end, we have resorted to the analytical approximations for the equation of state and radial distribution function of the fluid previously set up by Santos and López de Haro [6]. Over the fractional dimensionality range explored, the so-called “residual multiparticle entropy” (RMPE), obtained as the difference between the excess and pair entropies, shows a behavior utterly similar to that exhibited for integer one, two and three dimensions. Hence, on a phenomenological continuity basis, we surmise that hard spheres undergo an “ordering” transition even in a space with fractional dimensions, which may well anticipate a proper thermodynamic fluid-to-solid phase transition. This can serve as a motivation for future research.

We found that the packing fraction loci of minimum and vanishing RMPE show a monotonic decreasing behavior as a function of the dimensionality; this result is coherent with the magnification of excluded-volume effects produced by increasing spatial dimensionalities and, correspondingly, with a gradual shift of the ordering transition threshold to lower and lower packing fractions. However, it also turns out that the minimum value of the RMPE exhibits a non-monotonic behavior, attaining a minimum at the fractional dimensionality d=2.38. For this value of *d*, the relative entropic weight of more-than-two-particle correlations reaches, in the “gas-like” regime, its maximum absolute value.

A quasi-universal scaling of the RMPE over its minimum value in the neighborhood of the sign-crossover point was observed, thus suggesting that the properties of the local ordering phenomenon should not sensitively depend on the spatial dimensionality.

Finally, it must be stressed that the so-called “spreading dimension” $d_{l}$ is limited to values less than or equal to 2 [30,31]. In Ref. [5], the spreading dimension was identified as the relevant dimension of a fractal hard-sphere fluid on the incipient percolation cluster in a two-dimensional embedding space (D=2), in which case $d_{l}=1.67659\ldots$. If the (integer) value of *D* is increased beyond 2, then the value of $d_{l}$ increases as well, but for D≥6 the limiting value is $d_{l}=2$ [30]. To reach non-integer dimensions d>2, a completely new realization of the fractal hard-sphere fluid, which cannot be based on the percolation cluster as a configuration space [5], should be found. Therefore, the physical relevance of our results for non-integer dimensions larger than d=2 is presently unknown.

## Abbreviations

RMPE	Residual Multiparticle Entropy
MC	Monte Carlo
PY	Percus–Yevick
